# Comparative Evaluation of Medial Parapatellar and Transpatellar Tendon Approaches for Intramedullary Interlocking Nailing of Tibial Shaft Fractures: A Prospective Comparative Study

**DOI:** 10.7759/cureus.115761

**Published:** 2026-09-04

**Authors:** Vipan Kumar, Kautilya Kautilya, Ajay Pal Singh Sehrawat, Arun V Patial, Lokesh Thakur

**Affiliations:** 1 Department of Orthopaedics, Dr. Rajendra Prasad Government Medical College, Kangra, IND

**Keywords:** intramedullary nailing, knee joint, patellar ligament, range of motion, tibial fractures

## Abstract

Introduction

Intramedullary (IM) interlocking nailing is the standard treatment for displaced tibial shaft fractures. Our study aimed to compare postoperative pain, functional recovery, and complication profiles between the medial parapatellar and transpatellar tendon approaches in patients undergoing IM interlocking nailing for tibial shaft fractures.

Materials and methods

This prospective comparative study included 60 patients with tibial shaft fractures who underwent IM interlocking nailing; 30 patients each were allocated to the transpatellar tendon group and the medial parapatellar group. The demographic and clinical characteristics were recorded preoperatively. Postoperative outcomes included Visual Analog Scale (VAS) pain scores, knee range of motion (ROM), ankle ROM, operative duration, and postoperative complications. The patients were evaluated on postoperative days 14, 28, and 84. Continuous variables were compared using the independent-samples t-test, categorical variables using the chi-square test or Fisher's exact test, and effect sizes were calculated using Cohen's d. Statistical significance was set at p < 0.05.

Results

The mean age was comparable between the transpatellar (41.00 ± 13.82 years) and medial parapatellar (41.11 ± 13.30 years) groups (p = 0.975). Postoperative VAS scores progressively decreased in both groups, without significant differences on day 14 (6.55 ± 1.23 vs. 6.03 ± 1.16; p = 0.097), day 28 (4.10 ± 1.22 vs. 3.57 ± 0.93; p = 0.064), and day 84 (1.81 ± 0.94 vs. 1.43 ± 0.77; p = 0.091). Knee ROM improved significantly in both groups; however, on day 84, the medial parapatellar group demonstrated significantly greater knee ROM than the transpatellar group (118.33 ± 5.30° vs. 111.29 ± 8.06°; p < 0.001; Cohen's d = 1.03). Ankle ROM was comparable throughout follow-up, with no statistically significant differences (day 84: 24.66 ± 1.29° vs. 23.87 ± 3.08°; p = 0.203). Postoperative complications were also comparable between the groups, although anterior knee pain occurred more frequently after the transpatellar approach (26.7% vs. 10.0%).

Conclusion

Both surgical approaches achieved satisfactory postoperative outcomes following IM interlocking nailing for tibial shaft fractures. However, the medial parapatellar approach resulted in significantly superior recovery of knee ROM while providing comparable postoperative pain relief, ankle function, operative duration, and complication rates. Therefore, preservation of the patellar tendon may facilitate improved early functional recovery without compromising surgical safety, making the medial parapatellar approach a valuable alternative for the surgical management of tibial shaft fractures.

## Introduction

Tibial shaft fractures are among the most common long-bone injuries encountered in orthopedic trauma practice, primarily resulting from high-energy mechanisms, such as road traffic accidents and sports-related injuries, as well as low-energy falls in older adults [1]. Because the tibia has limited soft tissue coverage along its anteromedial surface, these fractures are associated with a substantial risk of complications, including delayed union, nonunion, infection, malalignment, and impaired functional recovery [1]. Intramedullary (IM) interlocking nailing is widely regarded as the standard treatment for displaced tibial shaft fractures because it provides stable fixation while preserving fracture biology, thereby allowing early mobilization and satisfactory fracture healing [2].

Despite the widespread use of IM nailing, the optimal surgical approach for nail insertion remains controversial. The conventional transpatellar tendon approach offers straightforward access to the tibial entry point; however, it has frequently been associated with postoperative anterior knee pain, difficulty kneeling, and tendon-related morbidity [3]. Alternatively, the medial parapatellar approach avoids splitting the patellar tendon and has been proposed to reduce soft tissue trauma while facilitating improved visualization of the tibial entry point [3,4]. Previous studies have reported conflicting findings regarding postoperative pain, knee function, range of motion (ROM), and complication rates between these two approaches, with no clear consensus on the superior technique [3-6]. Consequently, the selection of the surgical approach often depends on the surgeon’s preference and experience rather than robust clinical evidence.

Assessment of postoperative outcomes, such as pain intensity, knee and ankle ROM, and procedure-related complications, is essential for determining the functional effectiveness of different surgical approaches [1-6]. Comparative evaluation of these parameters can provide clinically relevant evidence to guide surgical decision-making and optimize patient recovery following tibial IM interlocking nailing. Therefore, further studies comparing the functional outcomes of the transpatellar and medial parapatellar approaches are warranted to establish evidence-based recommendations for clinical practice.

The primary objective of this study was to compare postoperative functional outcomes, including pain intensity measured using the Visual Analog Scale (VAS) and knee ROM, between the medial parapatellar and transpatellar tendon approaches in patients undergoing IM interlocking nailing for tibial shaft fractures. The secondary objective was to compare ankle ROM, operative characteristics, and postoperative complications associated with the two surgical approaches to determine their overall clinical effectiveness and safety.

## Materials and methods

Study design and setting

This prospective, comparative, observational study was conducted in the Department of Orthopaedics at Dr. Rajendra Prasad Government Medical College, Kangra, India, between August 2024 and October 2025. The study protocol adhered to the principles of the Declaration of Helsinki and was approved by the Institutional Ethics Committee (HFW-HDRPGMC/Ethics/2024/61, dated May 25, 2024) before the commencement of the study. Written informed consent was obtained from all participants before enrolment. Patients presenting with tibial shaft fractures who fulfilled the eligibility criteria and required IM interlocking nailing as part of routine clinical management were recruited during the study period. The investigators had no role in the selection of the surgical approach or the allocation of patients to either treatment group. The authors were involved only in prospective data collection, postoperative clinical evaluation, follow-up, and statistical analysis.

Sample size estimation

The sample size was estimated using G*Power software version 3.1.9.7 (Heinrich Heine University Düsseldorf, Düsseldorf, Germany). An a priori power analysis was performed to compare the two independent groups. Based on a previously published reference study that demonstrated a large effect size (Cohen's d = 0.80) for the primary outcome, a statistical power of 80% (β = 0.20) and a two-sided alpha level of 5% (α = 0.05) were considered [3]. The minimum required sample size was 27 participants per group. To compensate for an anticipated 10% attrition during follow-up, the sample size was increased to 30 participants per group, resulting in a final sample of 60 participants.

Participant selection

Adult patients diagnosed with isolated tibial shaft fractures that required surgical fixation with IM interlocking nailing were screened for eligibility. Patients who fulfilled the inclusion criteria and provided written informed consent were consecutively enrolled. Patients with pathological fractures, previous surgery involving the affected tibia, associated ipsilateral lower limb fractures affecting postoperative rehabilitation, severe neurovascular injuries, active infection at the operative site, or incomplete follow-up were excluded from the study.

Surgical approach selection

The surgical approach was selected by the treating orthopaedic surgeon as part of routine clinical decision-making and was not determined by randomization, stratification, blocking, or a predefined allocation algorithm. Surgeons considered the clinical presentation and fracture characteristics, together with their individual experience and preference, when selecting either the transpatellar tendon or medial parapatellar approach. The investigators did not participate in treatment allocation. Although fracture type and other recorded baseline characteristics were compared between groups, the study did not prospectively collect or formally quantify all factors that may have influenced the surgeon's choice of approach, including potentially unmeasured aspects of fracture severity. Therefore, the possibility of selection bias and confounding by indication cannot be excluded.

Surgical procedure

All surgical procedures were performed by experienced orthopaedic surgeons using standard operative protocols under appropriate regional or general anesthesia. The patients were positioned supine on a radiolucent operating table, with fluoroscopic guidance used throughout the procedure. Closed reduction was attempted whenever feasible before nail insertion. In the transpatellar tendon approach, a skin incision was made in the midline, extending across the patellar tendon, and the tendon was longitudinally split to gain access to the tibial entry point. Following guidewire insertion under fluoroscopic guidance, sequential reaming was performed when indicated, followed by the insertion of an appropriately sized tibial IM interlocking nail. Proximal and distal locking screws were inserted according to standard operative principles [3].

In the medial parapatellar approach, a longitudinal skin incision was made just medial to the patellar tendon. The tendon was gently retracted laterally without splitting, thereby exposing the proximal tibial entry point while preserving tendon integrity. Subsequent guidewire placement, canal preparation, nail insertion, and proximal and distal locking were performed using the same standardized technique employed in the transpatellar group [3]. To minimize performance bias, identical implant systems, reduction techniques, fluoroscopic guidance, and postoperative rehabilitation protocols were used in both study groups.

Postoperative management and follow-up

Standard postoperative care, including analgesics, IV antibiotics, thromboprophylaxis when indicated, and early mobilization, was provided uniformly to all patients. Active and passive knee and ankle ROM exercises were initiated according to institutional rehabilitation protocols. Progressive weight-bearing was permitted depending on fracture stability and radiographic healing. Patients were reviewed at 14, 28, and 84 days after surgery. During each follow-up visit, pain intensity, knee ROM, ankle ROM, wound condition, and postoperative complications were assessed using standardized clinical examination protocols.

Outcome measures

The primary outcome measures were postoperative pain intensity and knee function. Pain intensity was evaluated using the VAS, with higher scores indicating greater pain severity. Functional recovery was assessed by measuring active knee ROM in degrees using a standard universal goniometer. The secondary outcome measures included ankle ROM, operative duration, and postoperative complications, such as anterior knee pain and scar-related morbidity. Baseline demographic characteristics, including age, sex, mechanism of injury, fracture type, and associated comorbidities, were also recorded to assess comparability between the study groups.

Data collection

Demographic, clinical, operative, and postoperative data were recorded by investigators who were not involved in the statistical analysis. ROM measurements were performed using identical measurement techniques at each follow-up visit to ensure consistency. Pain scores and complication profiles were documented during each scheduled clinical review.

Statistical analysis

Statistical analysis was performed using IBM SPSS Statistics for Windows (version 25.0; IBM Corp., Armonk, New York, USA). Data normality was evaluated using the Shapiro-Wilk test. Continuous variables were expressed as mean ± SD, whereas categorical variables were presented as frequencies and percentages. Continuous variables were compared between the two study groups using the independent-samples t-test, while categorical variables were analyzed using the chi-square test or Fisher's exact test, as appropriate. The magnitude of differences between the groups was quantified using Cohen's d effect size. All statistical tests were two-tailed, and statistical significance was set at p < 0.05.

Because treatment allocation was non-randomized, between-group comparisons were interpreted as unadjusted associations rather than causal effects. Baseline demographic and clinical characteristics were compared between the two groups to identify potential differences that could influence postoperative outcomes. Given the observational design and limited sample size, residual confounding from measured or unmeasured factors could not be completely excluded.

## Results

Sixty patients were included in the study, with 30 (50.0%) patients in the transpatellar tendon group and 30 (50.0%) patients in the medial parapatellar group. The mean age was comparable between the transpatellar (41.00 ± 13.82 years) and medial parapatellar (41.11 ± 13.30 years) groups (p = 0.975). The transpatellar group comprised 26 (86.7%) males and 4 (13.3%) females, whereas the medial parapatellar group included 18 (60.0%) males and 12 (40.0%) females, demonstrating a statistically significant difference in sex distribution (p = 0.041). Road traffic accidents were the most frequent mechanism of injury in both groups, affecting 21 (70.0%) patients in the transpatellar group and 12 (40.0%) patients in the medial parapatellar group, followed by falls in 8 (26.7%) and 16 (53.3%) patients, respectively. The distribution of fracture types, comorbidities, and operative duration was comparable between the groups (p > 0.05). Postoperative complications were observed in a minority of patients, with anterior knee pain reported in 8 (26.7%) patients in the transpatellar group and 3 (10.0%) patients in the medial parapatellar group, while scar-related complications occurred in 2 (6.7%) and 5 (16.7%) patients, respectively. No postoperative complications were observed in 20 (66.7%) and 22 (73.3%) patients in the transpatellar and medial parapatellar groups, respectively. Overall, the two groups were comparable in most baseline characteristics (Table 1).

**Table 1:** Demographic and clinical characteristics of patients in the transpatellar and medial parapatellar groups. Data are presented as mean ± SD for continuous variables and number (percentage) for categorical variables. Intergroup comparisons were performed using the independent-samples t-test for continuous variables and the chi-square test for categorical variables. *p < 0.05 was considered statistically significant. χ² = chi-square statistic.

Variables	Category	Transpatellar (n = 30), n (%)	Parapatellar (n = 30), n (%)	Test value	p-value
Age (years)	Mean ± SD	41.00 ± 13.82	41.11 ± 13.30	t = -0.03	0.975
Sex	Male	26 (86.7)	18 (60.0)	χ² = 4.18	0.041*
	Female	4 (13.3)	12 (40.0)		
Mode of injury	Road traffic accident	21 (70.0)	12 (40.0)	χ² = 5.45	0.065
	Fall	8 (26.7)	16 (53.3)		
	Twisting injury	1 (3.3)	2 (6.7)		
Type of fracture	Transverse	8 (26.7)	9 (30.0)	χ² = 2.39	0.664
	Spiral	12 (40.0)	7 (23.3)		
	Oblique	4 (13.3)	7 (23.3)		
	Comminute	4 (13.3)	4 (13.3)		
	Butterfly	2 (6.7)	3 (10.0)		
Comorbidity	Hypertension	5 (16.7)	4 (13.3)	χ² = 2.31	0.510
	Diabetes mellitus	2 (6.7)	0 (0.0)		
	Others	2 (6.7)	2 (6.7)		
	None	21 (70.0)	24 (80.0)		
Operating time	< 60 min	26 (86.7)	23 (76.7)	χ² = 0.45	0.505
	≥ 60 min	4 (13.3)	7 (23.3)		
Complications	Anterior knee pain	8 (26.7)	3 (10.0)	χ² = 3.65	0.161
	Scar	2 (6.7)	5 (16.7)		
	None	20 (66.7)	22 (73.3)		

The VAS pain scores progressively decreased in both groups during the study period. On postoperative day 14, the mean VAS score was 6.55 ± 1.23 in the transpatellar group and 6.03 ± 1.16 in the medial parapatellar group (p = 0.097). On day 28, the respective scores were 4.10 ± 1.22 and 3.57 ± 0.93 (p = 0.064), while by day 84, they had decreased to 1.81 ± 0.94 and 1.43 ± 0.77 (p = 0.091). Although the pain scores remained consistently lower in the medial parapatellar group, the differences were not statistically significant at any follow-up interval (Table 2).

**Table 2:** Comparison of postoperative pain and functional outcomes between the transpatellar and medial parapatellar groups during follow-up. Data are presented as mean ± SD. Intergroup comparisons were performed using the independent-samples t-test, and effect sizes were calculated using Cohen's d. *p < 0.05 was considered statistically significant. VAS: Visual Analog Scale; ROM: Range of motion.

Parameter	Timepoint	Transpatellar (Mean ± SD)	Medial parapatellar (Mean ± SD)	Test value (t)	p-value	Cohen's d
VAS score	Day 14	6.55 ± 1.23	6.03 ± 1.16	1.69	0.097	0.44
	Day 28	4.10 ± 1.22	3.57 ± 0.93	1.89	0.064	0.49
	Day 84	1.81 ± 0.94	1.43 ± 0.77	1.72	0.091	0.45
Knee ROM (°)	Day 14	87.10 ± 13.20	89.83 ± 11.40	-0.86	0.394	0.22
	Day 28	105.16 ± 12.34	108.33 ± 11.16	-1.04	0.302	0.27
	Day 84	111.29 ± 8.06	118.33 ± 5.30	-3.99	<0.001*	1.03
Ankle ROM (°)	Day 14	18.55 ± 4.50	19.66 ± 5.16	-0.89	0.378	0.23
	Day 28	22.10 ± 4.43	23.45 ± 3.02	-1.39	0.169	0.36
	Day 84	23.87 ± 3.08	24.66 ± 1.29	-1.29	0.203	0.33

Knee ROM improved significantly over time in both groups. On postoperative day 14, the mean knee ROM was 87.10 ± 13.20° in the transpatellar group and 89.83 ± 11.40° in the medial parapatellar group (p = 0.394). On day 28, the corresponding values were 105.16 ± 12.34° and 108.33 ± 11.16° (p = 0.302). At the final follow-up on day 84, patients treated through the medial parapatellar approach achieved significantly greater knee ROM (118.33 ± 5.30°) than those treated through the transpatellar approach (111.29 ± 8.06°), with a highly significant difference (p < 0.001) and a large effect size (Cohen's d = 1.03) (Table 2).

Ankle ROM also demonstrated progressive improvement in both groups throughout the follow-up period. On postoperative day 14, the mean ankle ROM was 18.55 ± 4.50° in the transpatellar group and 19.66 ± 5.16° in the medial parapatellar group (p = 0.378). On day 28, the values increased to 22.10 ± 4.43° and 23.45 ± 3.02°, respectively (p = 0.169), and by day 84, they reached 23.87 ± 3.08° and 24.66 ± 1.29°, respectively (p = 0.203). No statistically significant differences were identified between the groups at any postoperative assessment (Table 2).

Overall, the medial parapatellar approach demonstrated superior recovery of knee function at the final follow-up while providing postoperative pain relief, ankle mobility, operative duration, and complication rates comparable to those achieved with the transpatellar tendon approach. The temporal changes in the VAS score, knee ROM, and ankle ROM are illustrated in Figure 1.

**Figure 1:**
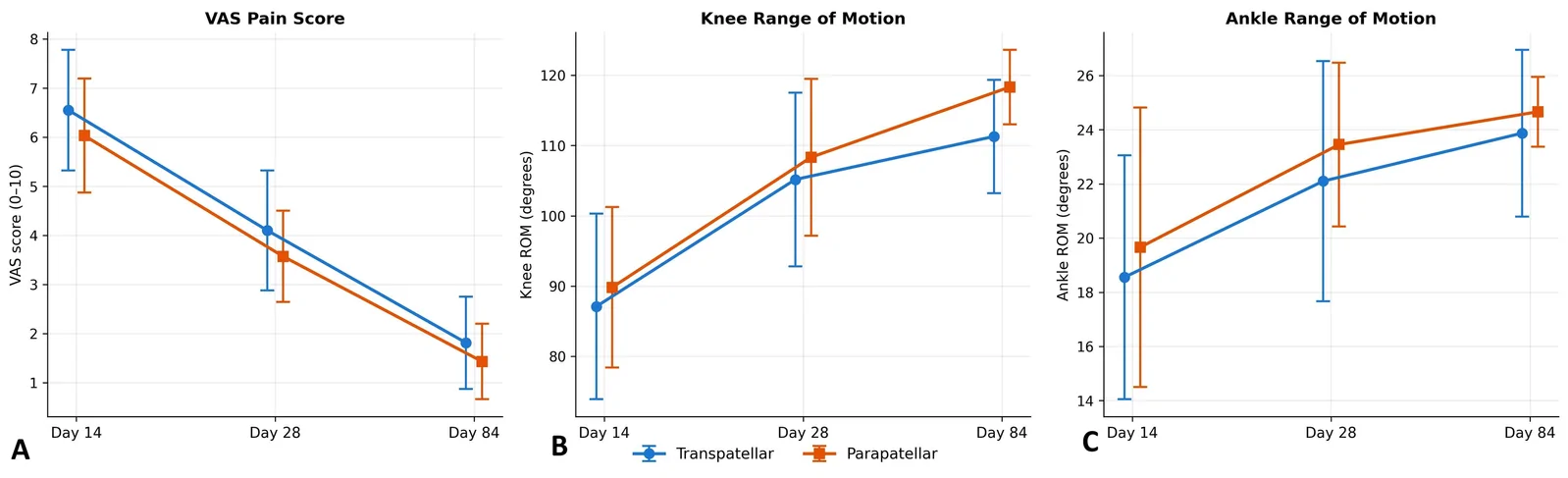
Longitudinal changes following transpatellar and medial parapatellar intramedullary nailing for tibial shaft fractures: (A) VAS pain score, (B) knee ROM, and (C) ankle ROM. The figure illustrates longitudinal changes in postoperative VAS pain scores, knee ROM, and ankle ROM on postoperative days 14, 28, and 84 in the transpatellar and medial parapatellar groups. VAS: Visual Analog Scale; ROM: Range of motion.

## Discussion

IM interlocking nailing remains the preferred treatment for displaced tibial shaft fractures because it provides stable fixation while preserving the biological environment necessary for fracture healing [2]. However, the choice of entry approach continues to be debated owing to its potential influence on postoperative pain, functional recovery, and patient satisfaction. The present prospective comparative study demonstrated that, although both the medial parapatellar and transpatellar tendon approaches produced comparable postoperative pain relief, ankle function, operative characteristics, and complication rates, the medial parapatellar approach resulted in significantly better knee ROM at the final follow-up. These findings suggest that patellar tendon preservation may contribute to improved early functional recovery without compromising surgical safety.

Although most recorded baseline characteristics were comparable between the groups, a significant difference in sex distribution was observed, with a higher proportion of males in the transpatellar group. Therefore, the groups cannot be considered fully balanced at baseline, and the unadjusted comparison of postoperative outcomes, including knee ROM, may be subject to residual confounding. The observed difference in knee ROM should consequently be interpreted cautiously and not attributed solely to the surgical approach. The predominance of road traffic accidents observed in the present study is also consistent with the established epidemiology of tibial shaft fractures reported in developing countries, where high-energy trauma remains the leading cause of these injuries [7,8].

Postoperative pain progressively decreased in both groups throughout the follow-up period, with no statistically significant intergroup differences. Nevertheless, patients who underwent the medial parapatellar approach consistently demonstrated lower mean pain scores at each evaluation. Although the differences did not reach statistical significance, the moderate effect sizes observed suggest that tendon preservation may provide clinically meaningful benefits during the early postoperative period. The transpatellar approach requires splitting of the patellar tendon, which may result in additional tendon trauma, localized inflammation, and irritation during knee flexion, thereby contributing to persistent anterior knee discomfort. In contrast, the medial parapatellar approach avoids direct violation of the tendon, potentially reducing soft tissue injury while providing access to the proximal tibial entry point [4]. The use of tendon-sparing or patellar-subluxation techniques to facilitate access to the proximal tibial entry point has also been described by Tornetta P 3rd and Collins E [9]. Similarly, Toivanen JA et al. [10] reported lower postoperative anterior knee pain following parapatellar nail insertion, supporting the biological rationale observed in the present study.

The most important finding of the present investigation was the significantly greater knee ROM achieved in the medial parapatellar group at the 84-day follow-up. Preservation of the patellar tendon and extensor mechanism likely facilitates earlier rehabilitation by reducing pain during active knee movement and minimizing postoperative scar formation around the tendon [3]. Furthermore, improved visualization of the proximal tibial entry point through medial retraction of the patellar tendon may facilitate more accurate nail insertion with less iatrogenic soft tissue trauma [4]. Similar improvements in postoperative knee function have been described by several investigators, including Keating JF et al. [11], who demonstrated superior functional recovery with tendon-sparing approaches, particularly during the early postoperative period. Similarly, Khan MN et al. [3] reported improved knee mobility and reduced functional limitations with the medial parapatellar approach, reinforcing the findings of the present study. Enhanced knee motion is particularly relevant because restoration of knee mobility is a major determinant of patient satisfaction, return to work, and overall quality of life following tibial fracture fixation.

Ankle ROM improved steadily in both groups without significant differences. This finding is expected because ankle rehabilitation primarily depends on fracture stability, soft tissue recovery, and postoperative physiotherapy rather than the proximal surgical approach used for nail insertion [12]. As both groups underwent identical fixation techniques, postoperative rehabilitation protocols, and weight-bearing progression, comparable ankle recovery was anticipated. These findings indicate that the choice of entry approach mainly influences local knee-related outcomes rather than distal joint function.

Although postoperative complications were comparable between the two groups, anterior knee pain was numerically more frequent following the transpatellar approach. Although this difference was not statistically significant, the finding is clinically relevant because anterior knee pain remains one of the most common complications of tibial IM nailing. This finding is consistent with the systematic review by Parikh S et al. [13], who reported that tendon-sparing approaches generally demonstrated improved patient-reported outcomes and a lower incidence of anterior knee pain than conventional infrapatellar techniques, although evidence specifically comparing medial parapatellar and transpatellar approaches remains limited. Soraganvi PC et al. [14] reported that anterior knee pain is strongly associated with nail prominence greater than 5 mm from the anterior tibial cortex and a proximally positioned nail tip, emphasizing that factors related to surgical technique and implant positioning contribute substantially to postoperative knee discomfort. Their findings suggest that careful attention to entry point selection and nail positioning may reduce the incidence of anterior knee pain after tibial nailing.

Song SY et al. [15] reported that anterior knee pain remained a frequent complication even after the medial paratendinous approach, with moderate to severe pain observed in 28% of patients. They further demonstrated that greater superior nail prominence and increased nail apex distance were significantly associated with more severe anterior knee pain and poorer functional outcomes, highlighting that implant positioning, in addition to the surgical approach, plays a critical role in postoperative recovery. Similarly, Lu K et al. [16], in a meta-analysis of 15 studies involving 1,396 patients, demonstrated that the surgical approach significantly affected postoperative pain and knee function, with approaches that minimized periarticular soft tissue trauma generally producing superior clinical outcomes.

Overall, the findings of the present study support a growing body of evidence favoring the medial parapatellar approach as an effective alternative to the conventional transpatellar tendon approach. The improved knee function observed without an increase in operative time or complications suggests that preservation of the extensor mechanism may provide meaningful functional advantages while maintaining equivalent surgical efficacy.

Clinical implications and limitations

The findings of this study have important clinical implications for orthopaedic surgeons managing tibial shaft fractures. The medial parapatellar approach may be considered a preferred technique in patients in whom rapid restoration of knee function and early rehabilitation are prioritized, particularly in physically active individuals. By preserving the patellar tendon, this approach may facilitate improved postoperative mobility without increasing surgical complexity or complication rates.

Nevertheless, this study has several limitations. Its single-center design, modest sample size, and short follow-up period of 84 days may limit generalizability and preclude the assessment of long-term functional outcomes, chronic anterior knee pain, fracture union, and implant-related complications. The non-randomized, surgeon-driven allocation of the surgical approach introduces the possibility of selection bias and confounding by indication. Although most recorded baseline characteristics were comparable, the significant difference in sex distribution and the possibility of unmeasured differences in clinical or fracture-related factors mean that residual confounding cannot be excluded. Additionally, the use of a previous retrospective single-center study to inform the sample-size calculation and as supporting literature for the present findings may introduce some degree of circularity; therefore, confirmation by independent studies in different populations and settings is warranted. In addition, repeated outcomes were analyzed using separate independent-samples t-tests at each follow-up rather than a repeated-measures approach, which does not account for within-subject correlation or multiple comparisons. Accordingly, the longitudinal patterns shown in Figure 1 are descriptive, and the observed differences should be interpreted cautiously. Future multicenter randomized studies using appropriate longitudinal analyses and longer follow-up are warranted.

## Conclusions

Both the medial parapatellar and transpatellar tendon approaches were associated with satisfactory clinical outcomes following IM interlocking nailing for tibial shaft fractures. The medial parapatellar group demonstrated greater knee ROM at the 84-day assessment, while postoperative pain, ankle function, operative duration, and complication rates were comparable between the groups. However, given the non-randomized allocation and the use of separate between-group comparisons at each follow-up time point without repeated-measures analysis, these findings should be interpreted cautiously. Further randomized studies using appropriate longitudinal analyses and longer follow-up are warranted.
